# Developing a genetic approach to investigate the mechanism of mitochondrial competence for DNA import

**DOI:** 10.1016/j.bbabio.2008.11.001

**Published:** 2009-05

**Authors:** Frédérique Weber-Lotfi, Noha Ibrahim, Pierre Boesch, Anne Cosset, Yuri Konstantinov, Robert N. Lightowlers, André Dietrich

**Affiliations:** a Institut de Biologie Moléculaire des Plantes, CNRS and Université Louis Pasteur, 12 rue du Général Zimmer, 67084 Strasbourg, France; b School of Neurology, Neurobiology and Psychiatry, Medical School, University of Newcastle upon Tyne, Framlington Place, Newcastle upon Tyne, NE2 4HH, UK; c Institute of Plant Physiology and Biochemistry, Russian Academy of Science, Ul. Lermontova 132, Irkutsk 664033, Russia

**Keywords:** Mitochondrion, DNA import competence, Membrane transport, Yeast mutant, VDAC, Mitochondrial transfection

## Abstract

Mitochondrial gene products are essential for the viability of eukaryote obligate aerobes. Consequently, mutations of the mitochondrial genome cause severe diseases in man and generate traits widely used in plant breeding. Pathogenic mutations can often be identified but direct genetic rescue remains impossible because mitochondrial transformation is still to be achieved in higher eukaryotes. Along this line, it has been shown that isolated plant and mammalian mitochondria are naturally competent for importing linear DNA. However, it has proven difficult to understand how such large polyanions cross the mitochondrial membranes. The genetic tractability of *Saccharomyces cerevisae* could be a powerful tool to unravel this molecular mechanism. Here we show that isolated *S. cerevisiae* mitochondria can import linear DNA in a process sharing similar characteristics to plant and mammalian mitochondria. Based on biochemical data, translocation through the outer membrane is believed to be mediated by voltage-dependent anion channel (VDAC) isoforms in higher eukaryotes. Both confirming this hypothesis and validating the yeast model, we illustrate that mitochondria from *S. cerevisiae* strains deleted for the VDAC-1 or VDAC-2 gene are severely compromised in DNA import. The prospect is now open to screen further mutant yeast strains to identify the elusive inner membrane DNA transporter.

## 1 Introduction

On the basis of comparative genomics, mitochondria, organelles common to most eukaryotic species, are believed to have evolved from a metabolically-driven symbiotic event (see [1,2]). Several theories have been proposed [3,4], but perhaps the most widely accepted is that as the oxygen levels increased in the environment an anaerobic host engulfed an aerobic α-proteobacterium to relieve oxygen tension [1]. This endosymbiotic relationship was then cemented by the reductive loss of large parts of the endosymbiont genome. As has been well documented in the yeast *Saccharomyces cerevisiae*, DNA can be shown to move from the mitochondrion to the nucleus [5], but is it possible for DNA to be taken into the mitochondrion? This question is of great relevance to the human organelle, as there is currently no system for transfecting human mitochondria as a means of rescuing mutations of the mitochondrial genome (mtDNA) [6]. Mutations of this genome are known to underlie a series of rare, mainly muscle and neurological diseases but have been implicated in more common neurodegenerative disorders and the ageing process itself [7–9]. It is also of importance in plant genetics, where mitochondrial genetic information influences several traits of agronomical importance, including male sterility, plant vigor, chloroplast function and cross-compatibility [10]. Consequently, the ability to import DNA into mitochondria may have important therapeutic and economic potential.

It is well established that bacteria are able to laterally transfer DNA between species by a variety of methods [11], with some species of proteobacteria being able to import DNA directly from the environment (natural competence) [12,13]. DNA can be transported across membranes through the conjugation transport system or through the SpoIIIE/FtsK family of DNA transporters that facilitate chromosome segregation during bacterial division [14]. These are complex systems, but we are currently trying to determine whether mitochondria may have retained a cryptic or simplistic form of DNA transporter. Bioinformatic analysis has identified no candidate orthologues for any bacterial gene product implicated in DNA import. However, many plant mitochondrial genomes appear to be evolutionary mosaics of DNA sequences from various sources (nuclear, viral, chloroplastic and unknown) [15], suggesting the retention of DNA import competence, at least in these species. Consistent with this observation, Koulintchenko et al. [16] reported that linearised DNA was able to be taken up into isolated mitochondria from *Solanum tuberosum* and *Arabidopsis thaliana* and the imported DNA acted as a template for DNA and RNA synthesis. This competence has been exploited to show that mitochondria from both species contain the enzymes necessary to repair DNA (Boesch et al., submitted for publication). Surprisingly, although mammalian mtDNA is remarkably compact and shows little evidence of mosaicism, isolated rat and human mitochondria can also import linearised DNA, with the imported template being transcribed in a promoter-dependent manner, and the primary transcript being processed and matured [17].

What are the transporters and what is the exact molecular mechanism of mitochondrial competence? Original experiments with isolated plant and mammalian mitochondria pointed to a potential role of the voltage-dependent anion channel (VDAC) in DNA transport through the outer membrane [16,17], but no factors have been identified to date that mediate DNA transfer through the inner membrane, although candidates have been found. It is clear that to progress our knowledge of this process it is necessary to model mitochondrial competence in a system that is genetically tractable. Therefore, we have initiated studies on mitochondria isolated from the yeast, *S. cerevisiae*. Many strains are available that carry deletions in mitochondrial carrier proteins which could potentially play a role in DNA import. Further, by identifying candidate transporters through a biochemical approach, we expect to be able to delete components of the natural competence system, therefore confirming their role in this process. In the present paper, we report that mitochondria from *S. cerevisiae* can indeed import DNA in a facile manner. To show the potential of the genetic approach, we confirm the central role of VDAC in the import process by demonstrating that deletions of VDAC isoforms restrict import. Finally, we report the proteomic analysis of a complex that is bound by DNA, leading to the identification of candidate gene products which may be involved in import.

## 2 Materials and methods

### 2.1 Yeast strains

Four yeast (*S. cerevisiae*) strains were used: the parental *POR1*–*POR2* strain M3 (*MAT*a *lys2 his4 trp1 ade2 leu2 ura3*), the strain M22-2 (*Δpor1*) containing a deletion of most of the *POR1* gene [18], the strain M3-2 (*Δpor2*) containing a deletion of the *POR2* coding region [19] and the strain M22-2-1 (*Δpor1*–*Δpor2*) double mutant [19].

### 2.2 Isolation of mitochondria

Yeast cells were grown at 28 °C in YPGal medium (1% w/v yeast extract, 2% w/v peptone, 2% w/v galactose, pH 5.5) and yeast mitochondria were isolated essentially according to Daum et al. [20]. Cells were pelleted by centrifugation at 3000 ×*g* for 5 min, washed once with distilled water, resuspended in 3 mL/g (wet weight) DTT buffer (100 mM Tris–SO_4_, pH 9.4, 10 mM dithiothreitol) and shaken for 20 min at 30 °C. They were then washed once with sorbitol buffer (1.2 M sorbitol, 20 mM Hepes–KOH, pH 7.4) and resuspended at 250 mg of cell wet weight/mL in the same buffer containing 1 mg of zymolyase-100T (MP Biomedicals) per gram of cell wet weight. The suspension was incubated for 30 min at 30 °C with gentle shaking for conversion into spheroplasts and these were harvested by centrifugation for 5 min at 3000 ×*g*. From this point on, all steps were carried out at 4 °C. Spheroplasts were washed twice in sorbitol buffer containing 1 mM PMSF and resuspended at 150 mg of cell wet weight/mL in homogenization buffer (0.6 M sorbitol, 20 mM Hepes–KOH, pH 7.4) containing 1 mM PMSF. Homogenization was carried out by 20 strokes in a glass-Teflon potter. Mitochondria were recovered by two cycles of low speed (3000 ×*g*) and high speed (12,000 ×*g*) centrifugation with intermediate resuspension in homogenization buffer. Protein concentration was determined using the Bio-Rad Protein Assay (Bio-Rad laboratories). Cauliflower (*Brassica oleracea*) mitochondria were isolated using the method described for potato [16] and rat (*Rattus norvegicus*) mitochondria were prepared as previously described [17].

### 2.3 DNA substrates for mitochondrial import

The DNA substrate used for yeast or cauliflower mitochondrial import assays was the 2.3 kb linear plasmid (accession no. X13704) from maize (*Zea mays*) [21] or a smaller fragment of 519 bp corresponding to nucleotides 148–666 of the *orf1* in the CIRV (*Carnation Italian ringspot virus*) RNA (accession no. X85215) [22]. The 1060 bp probe used for import into rat mitochondria was described previously [17]. All these DNA substrates were PCR generated and radiolabeled as described [16].

### 2.4 Mitochondrial import assays

For yeast, a standard mitochondrial import of DNA was carried out in import buffer (0.6 M mannitol, 10 mM glutamate, 1 mM malate, 40 mM Tris–HCl, pH 7.25). The samples (200 μL) containing 1–5 ng of [^32^P]-labeled DNA (100–200,000 cpm) and an amount of mitochondria corresponding to 100 μg of proteins were incubated at 30 °C for 40 min. Following addition of 50 μg of DNase-I and 10 mM MgCl_2_, the incubation was continued for 20 min. Mitochondria were subsequently washed three times by resuspension in 1 mL washing buffer (0.6 M mannitol, 10 mM EDTA, 10 mM EGTA, 40 mM Tris–HCl, pH 7.25) and centrifugation at 11,000 ×*g* for 5 min. The final pellets were extracted with 200 μL of 1 mM EDTA, 1% w/v SDS, 10 mM Tris–HCl, pH 7.5 and 200 μL of phenol. The nucleic acids recovered in the aqueous phase were ethanol-precipitated in the presence of 0.2 M NaCl, fractionated by electrophoresis on a 1% w/v agarose gel and transferred onto a nylon membrane (Hybond-XL, Amersham/GE Healthcare) for autoradiography. In all cases, the efficiency of total mitochondrial nucleic acid extraction from the different samples following import was confirmed by comparing the ethidium bromide staining patterns. DNA import into cauliflower and rat mitochondria was run as previously described [16,17].

To produce mitoplasts after import, yeast mitochondria were centrifuged for 10 min at 11,000 ×*g*, resuspended in 100 μL of import buffer and subjected to hypotonic swelling by a 10-fold dilution in 20 mM Hepes–KOH, pH 7.4, followed by a 30 min incubation on ice. Resulting mitoplasts were re-isolated by centrifugation at 11,000 ×*g* for 15 min and resuspended in 200 μL import buffer prior to DNase-I treatment and nucleic acid extraction as above. For mock treatment, washing buffer was used instead of 20 mM Hepes–KOH. To check the generation of mitoplasts, aliquotes of the samples were assayed for matrix (citrate synthase) and intermembrane space (adenylate kinase) enzymatic markers. Citrate synthase and adenylate kinase activities were assayed as described by Shepherd and Garland [23] and McGregor et al. [24], respectively.

### 2.5 Membrane protein extraction and analysis

Mitochondrial pellets were freeze-thawed three times and resuspended (2 mg/mL) at 4 °C in 5 mM potassium phosphate, pH 7.2, 500 μM PMSF. The suspensions were maintained 10 min at 4 °C, sonicated five times during 10 s at 4 °C and centrifuged at 150,000 ×*g* for 15 min. The membrane pellets were used for SDS-PAGE (4% w/v acrylamide stacking gel and 15% w/v acrylamide separation gel). Gels were run at 30 mA and stained with Coomassie Blue. Some protein bands were cut out from the gel and their protein content was identified by mass spectrometry. For this, samples were digested in-gel with trypsin as described by Rabilloud et al. [25] and MALDI-TOF mass measurements were carried out on a Biflex III (Bruker-Daltonik GmbH, Bremen, Germany) matrix-assisted laser desorption/ionization time-of-flight mass spectrometer used in reflector positive mode. Monoisotopic peptide masses were assigned and the peak list transferred through MS BioTools™ to the search engine MASCOT (Matrix Science, London, UK). The data were searched against the NCBI non-redundant protein sequence database.

VDAC-1 was identified on the gel by immunodetection. For this, SDS-PAGE gels were blotted onto Immobilon-P membrane (Millipore) and western blot analysis was conducted according to standard procedures.

### 2.6 Analysis of import experiments by Blue Native PAGE

After two-fold scale DNA import experiments with yeast mitochondria (see above), organelle pellets were freeze-thawed three times, resuspended in 250 μL homogenization buffer (0.6 M sorbitol, 20 mM Hepes–KOH, pH 7.4) and sonicated three times during 5 s at 4 °C. The suspensions were centrifuged at 100,000 ×*g* for 15 min and the pellets were resuspended in 150 μL ACA750 buffer (750 mM amino dicaproic acid, 50 mM Bis–Tris pH 7.0, 0.5 mM EDTA, 1 μg/mL leupeptin, 1 mM PMSF, 0.1 μg/mL α_2_-macroglobulin). Complexes were solubilised by adding 1% w/v digitonin and pippeting for 30 min on ice. The suspensions were centrifuged at 100,000 ×*g* for 15 min at 4 °C and 5% v/v Serva Blue solution (5% w/v Serva Blue G250 in ACA750 buffer) was added to the supernatant. The resulting samples were fractionated on one-dimensional Blue Native gels as described by Giegé et al. [26]. The radiolabeled complexes were detected with a phosphorimager plate and their protein content was analysed by nano-LC-MSMS mass spectrometry.

## 3 Results and discussion

### 3.1 Isolated yeast mitochondria are competent for DNA uptake

**Fig. 1:**
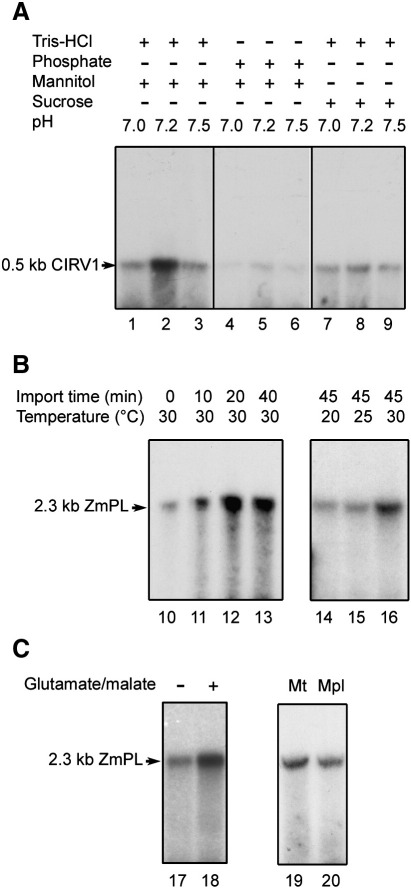
DNA can be imported into yeast mitochondria. (A) Testing different media. Labeled DNA representing a 0.5 kb fragment of CIRV *orf1* (0.5 kb CIRV1, see Materials and methods) was incubated for 40 min at 30 °C with isolated yeast (*S. cerevisiae*) mitochondria (parental strain M3, see Materials and methods) in media containing different combinations of buffer (Tris–HCl or potassium phosphate 40 mM, pH as indicated) and sugar (0.5 M sucrose or 0.6 M mannitol as indicated) prior to DNase-I digestion. Mitochondrial nucleic acids were subsequently extracted, fractionated by agarose gel electrophoresis and transferred onto a nylon membrane which was autoradiographed. (B) Time course and temperature dependence of DNA uptake. Labeled linear maize (*Zea mays*) 2.3 kb mitochondrial plasmid (2.3 kb ZmPL, see Materials and methods) was incubated for different times at 30 °C (lanes 10 to 13) or at different temperatures for 40 min (lanes 14 to 16) with yeast mitochondria in the Tris–HCl/mannitol medium. Uptake was analysed as in A. (C) DNA uptake is enhanced by glutamate/malate and the DNA reaches the matrix. Lanes 17 and 18: labeled maize 2.3 kb plasmid (2.3 kb ZmPL) was incubated with yeast mitochondria in the absence or presence of glutamate (10 mM) and malate (1 mM) in the Tris–HCl/mannitol medium for 40 min at 30 °C. Lanes 19 and 20: following incorporation of labeled maize 2.3 kb plasmid for 40 min, yeast mitochondria were mock-treated (Mt) or submitted to osmotic shock (Mpl) before DNase-I treatment. Intermembrane space and matrix marker enzymes were assayed with the same samples to assess efficiency of mitoplasting. Uptake was analysed as in A. Migration of the incorporated substrates is indicated (0.5 kb CIRV1 and 2.3 kb ZmPL).

Tests for DNA import into isolated *S. cerevisiae* mitochondria were developed using radioactively labeled, double-stranded substrates of 0.5 kb (a fragment of CIRV *orf1*) or 2.3 kb (the maize mitochondrial 2.3 kb linear plasmid) (Fig. 1A), as described in Materials and methods. Following co-incubation, mitochondrial incorporation was characterised by acquired resistance to extensive DNase-I digestion. Various experimental conditions were tested and DNA uptake into yeast mitochondria was obtained in a minimal medium containing an osmoticum and a buffer, as assessed by the recovery of DNase-resistant full length import substrate in the final organelle fraction. Dependence of the uptake on the size or sequence of the DNA was not observed with the two substrates tested, which was consistent with the data from previous import experiments using various DNA substrates with plant and mammalian mitochondria [16,17]. Higher levels of incorporation were obtained in a Tris–HCl/mannitol medium (Fig. 1A, lanes 1 to 3). The process was dependent on the pH, with an optimal efficiency around pH 7.2 (Fig. 1A, lanes 2, 5, 8). According to time course analyses, DNA incorporation into the mitochondrial fraction was progressive and reached a plateau after about 40 min (Fig. 1B, lanes 10 to 13). Analysis of the temperature dependence identified an optimum at 30 °C (Fig. 1B, lanes 14 to 16). Whereas respiratory substrates were not necessary for import, a significant increase was detected in the presence of glutamate and malate (Fig. 1C, lanes 17 and 18). The overall efficiency was in the range of 0.05 to 0.10 fmol of DNA incorporated into 100 μg of mitochondrial protein, comparable to previously estimated values for plant and mammalian mitochondria [16,17]. Following the observations made, general conditions for DNA import into isolated *S. cerevisiae* mitochondria were defined as 40 min at 30 °C in a 40 mM Tris–HCl, 0.6 M mannitol, 10 mM glutamate, 1 mM malate medium at pH 7.25.

DNA was potentially being protected from degradation through non-specific interactions with the outer membrane or by accessing the DNase-impermeable intermembrane space. To preclude this possibility, after import, mitochondria were subjected to an osmotic shock to disrupt the outer membrane before DNase treatment. Under the conditions used, about 95% of the intermembrane space marker adenylate kinase was lost (data not shown), implying efficient mitoplasting, whereas recovery of the matrix marker citrate synthase was close to 100%, showing that mitoplasts were not disrupted. Such a treatment had no significant influence on the DNase resistance of the incorporated DNA, as compared to a mock-treatment (Fig. 1C, lanes 19 and 20), implying that the DNA was protected by the mitochondrial inner membrane and thus had crossed both mitochondrial membranes.

These results are consistent with isolated yeast mitochondria retaining a natural mechanism for DNA import similar to that of isolated plant and mammalian mitochondria. This is an important progression. It has now been several years since the first report of natural competence of mitochondria [16]. This natural import has been used to explore general DNA maintenance processes but, without access to a genetically amenable system, it has proven extremely difficult to resolve and identify the underlying molecular mechanisms.

### 3.2 Mitochondrial competence is sensitive to VDAC effectors

**Fig. 2:**
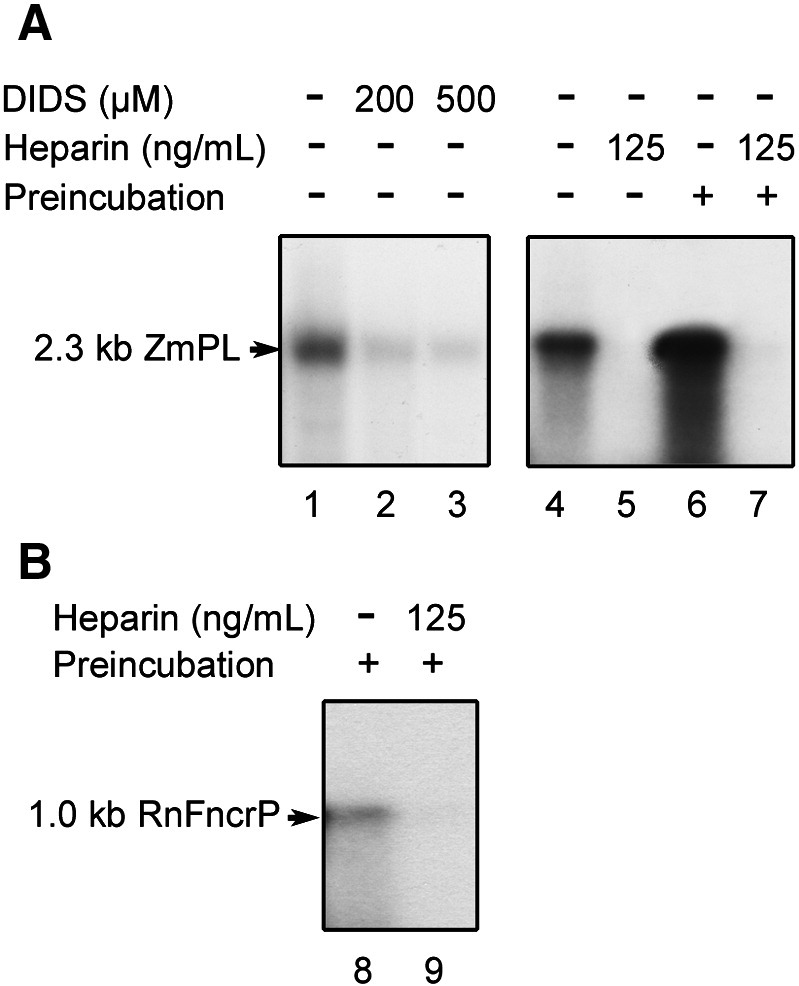
DNA import into plant and mammalian mitochondria is inhibited by VDAC effectors. (A) DIDS and heparin inhibit DNA uptake into plant mitochondria. Labeled maize 2.3 kb plasmid (2.3 kb ZmPL) was incubated for 45 min at 25 °C with isolated cauliflower (*Brassica oleracea*) mitochondria in the absence or presence of 4,4′-diisothiocyanatostilbene-2,2′-disulfonic acid (DIDS, lanes 1 to 3) or heparin (lanes 4 and 5) in standard plant DNA import conditions [16] prior to DNase-I digestion. As the DIDS stock solution was prepared in DMSO, the corresponding control assay (lane 1) was run in the presence of 0.5% v/v DMSO, which was representative for the amount of solvent introduced by the highest DIDS concentration used (500 μM). Uptake was analysed as in Fig. 1A. Lanes 6 and 7: cauliflower mitochondria were preincubated for 30 min in import conditions in the absence or presence of heparin, pelleted, resuspended in import medium and used for a standard import assay with labeled maize 2.3 kb plasmid. Uptake was analysed as in Fig. 1A. (B) Heparin inhibits DNA uptake into mammalian mitochondria. Rat (*Rattus norvegicus*) mitochondria were preincubated for 30 min in standard mammalian DNA import conditions [17] in the absence or presence of heparin; the organelles were then pelleted, resuspended in import medium and used for a standard import assay (80 min at 30 °C) with a 1 kb labeled DNA fragment corresponding to the non-coding region of the rat mitochondrial genome flanked by the tRNA^Phe^ and tRNA^Pro^ genes (1.0 kb RnFncrP, see Materials and methods). Uptake was analysed as in Fig. 1A. Migration of the incorporated substrates is indicated (2.3 kb ZmPL and 1.0 kb RnFncrP).

Based on the inhibitory effect of specific antibodies and of the effector Ruthenium Red, previous studies with plant (potato tuber) mitochondria suggested an involvement of the VDAC in DNA translocation through the outer membrane [16]. Interaction of the VDAC with the DNA is also plausible because it contains nucleotide-binding sites [27] and has been shown to bind tRNAs [28]. The idea was subsequently further supported by the sensitivity of DNA import into mammalian (rat liver and human cell) mitochondria to the semi-specific anion channel blocker 4,4′-diisothiocyanatostilbene-2,2′-disulfonic acid (DIDS) [17]. In further assays with the 2.3 kb maize plasmid, DIDS at a concentration of 200 μM also inhibited DNA incorporation into plant (cauliflower) organelles (Fig. 2A, lanes 2 and 3).

The polyanion heparin is another anion channel inhibitor, which can interact with the VDAC. Heparin added to the reaction medium at 125 ng/mL indeed abolished DNA import into cauliflower mitochondria (Fig. 2A, lanes 4 and 5). Interestingly, when the organelles were preincubated with heparin and washed, subsequent import of DNA was also fully inhibited (Fig. 2A, lanes 6 and 7), suggesting that heparin saturated binding sites on the VDAC or possibly on another upstream interacting protein on the outer membrane. The same effect was obtained with rat liver mitochondria (Fig. 2B, lanes 8 and 9).

**Fig. 3:**
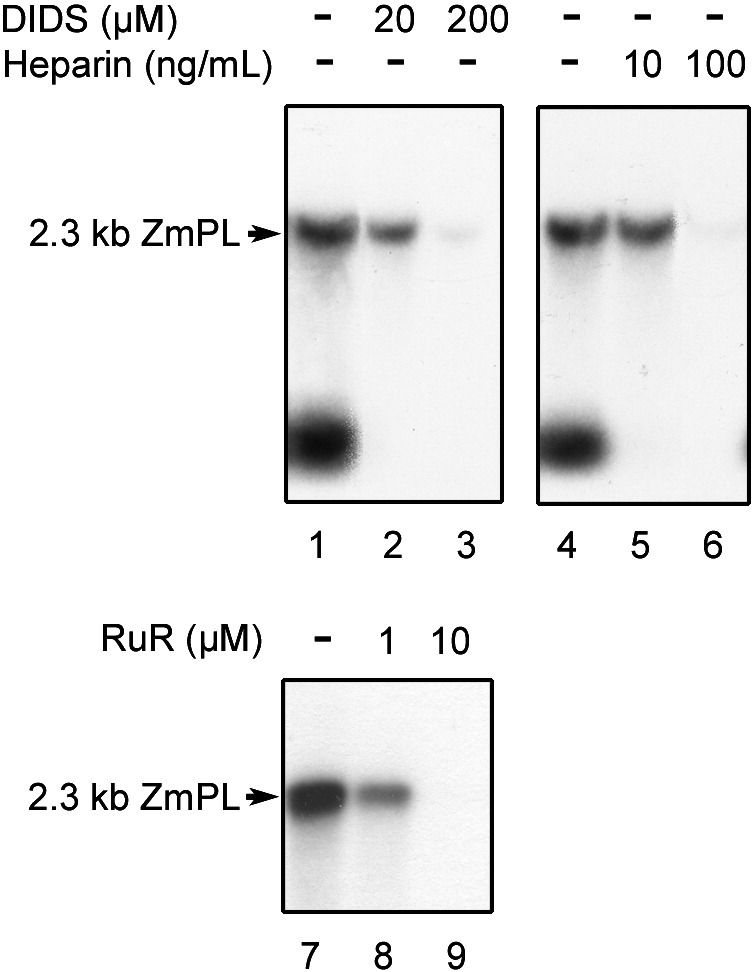
DNA import into yeast mitochondria is inhibited by VDAC effectors. Labeled maize 2.3 kb plasmid (2.3 kb ZmPL) was incubated for 40 min at 30 °C with isolated yeast (parental strain M3) mitochondria in the absence or presence of 4,4′-diisothiocyanatostilbene-2,2′-disulfonic acid (DIDS, lanes 1 to 3), heparin (lanes 4 to 6) or Ruthenium Red (RuR, lanes 7 to 9) in standard DNA import conditions prior to DNase-I digestion. As the DIDS stock solution was prepared in DMSO, the corresponding control assay (lane 1) was run in the presence of 0.2% v/v DMSO, which was representative for the amount of solvent introduced by the highest DIDS concentration used (200 μM). Uptake was analysed as in Fig. 1A. Migration of the incorporated substrate is indicated (2.3 kb ZmPL).

DNA uptake into yeast mitochondria showed a similar behavior in the presence of the above effectors. DIDS at 200 μM or heparin at 100 ng/mL completely inhibited the process. Both the signal corresponding to the imported full length substrate and the signal representative for the pool of small DNAse-I-generated fragments usually associated with the mitochondrial fraction at the end of the assay disappeared (Fig. 3, lanes 1 to 3 and 4 to 6). Also Ruthenium Red abolished DNA import into *S. cerevisiae* mitochondria in the same concentration range as in the case of plant organelles (Fig. 3, lanes 7 to 9). Biochemical data with the yeast system thus further point to DNA translocation through the outer membrane being mediated by the VDAC.

### 3.3 Mutation of individual VDAC genes impairs mitochondrial competence

**Fig. 4:**
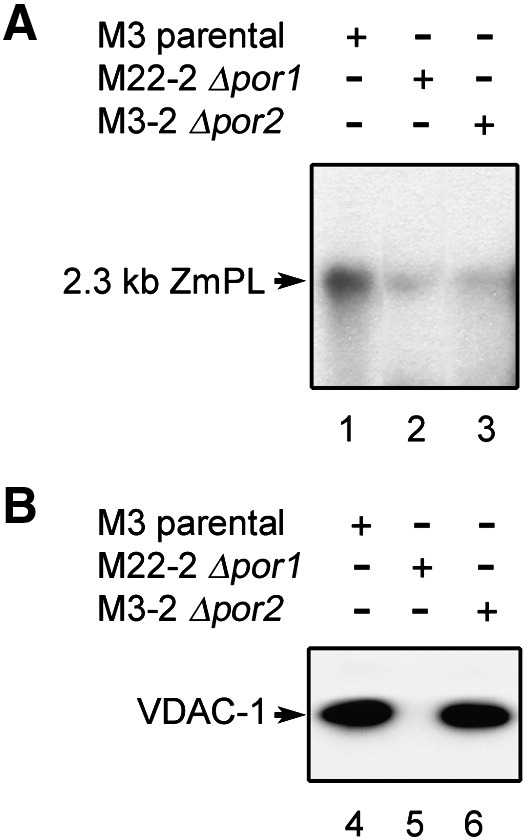
DNA import into mitochondria from yeast individual VDAC mutants is impaired. (A) DNA import assays. Labeled maize 2.3 kb plasmid (2.3 kb ZmPL) was incubated for 40 min at 30 °C in standard DNA import conditions with mitochondria isolated from the parental yeast strain M3, from the M22-2 *Δpor1* strain or from the M3-2 *Δpor2* strain. Following DNase-I digestion, uptake was analysed as in Fig. 1A. Migration of the incorporated substrate is indicated (2.3 kb ZmPL). (B) Western blot analysis of VDAC-1. Mitochondrial membrane proteins from the parental yeast strain M3, the M22-2 *Δpor1* strain and the M3-2 *Δpor2* strain were fractionated by SDS-PAGE and blotted. The blot was probed with an antiserum specific for yeast VDAC-1 and revealed by chemiluminescence with a peroxidase-conjugated secondary antibody. Migration of VDAC-1 is indicated.

The observations altogether indicated that yeast mitochondria are competent for DNA import in the same way as plant and mammalian organelles. Exploiting yeast genetic tools thus appeared to be a relevant approach to progress in the understanding of the translocation mechanism underlying such a process. As a first step, in line with the above biochemical studies, we analysed the import capacity of mitochondria isolated from strains mutated in VDAC genes. *S. cerevisiae* possesses two VDAC-encoding genes, *POR1* and *POR2* [19]. The originally identified *POR1* gene encodes VDAC-1, the major isoform. *POR2* encoding VDAC-2 was subsequently identified in a screen for genes which, upon overexpression, could functionally complement the defects of *POR1*-deleted strains, although its pore forming ability has been questioned [19,29]. Mitochondria isolated from the *Δpor1* strain M22-2 deleted for most of the *POR1* gene showed a strongly reduced DNA uptake capacity versus those from the control strain M3 (Fig. 4A, lanes 1 and 2). The absence of the VDAC-1 protein was assessed through western blot analysis with an antiserum specific for this isoform (Fig. 4B, lanes 4 and 5). Mitochondria from the *Δpor2* strain M3-2 lacking the coding sequence of the *POR2* gene had also lost most of their DNA import competence (Fig. 4A, lanes 1 and 3). The genetic tractability of *S. cerevisiae* thus allowed us to show that individual depletion of the genes encoding the VDAC isoforms severely restricts DNA import into yeast mitochondria. Strikingly, deletion of neither VDAC isoform was sufficient to completely prevent protection of some DNA following the import assay. In particular, the depletion in protected DNA that was seen repeatedly following the import assay with mitochondria from the *Δpor2* strain further questions the mechanism, since it has been suggested that VDAC-2, contrary to VDAC-1, may not normally form a channel [19]. This suggests that VDAC-1 alone is not sufficient to maintain maximal DNA uptake, although it is present at a normal level in the *Δpor2* strain (Fig. 4B, lane 6). Two ideas can be put forward to explain such a situation. The first is that, although the DNA would translocate through the channels formed by VDAC-1, VDAC-2 would have a major contribution to its recruitment and binding to the mitochondrial membrane. A second possibility is that VDAC-1 and VDAC-2 would form a “DNA import pore” as a heterodimer. The two hypotheses are not necessarily mutually exclusive.

### 3.4 DNA associates with a high molecular weight mitochondrial protein complex potentially containing the VDAC

**Fig. 5:**
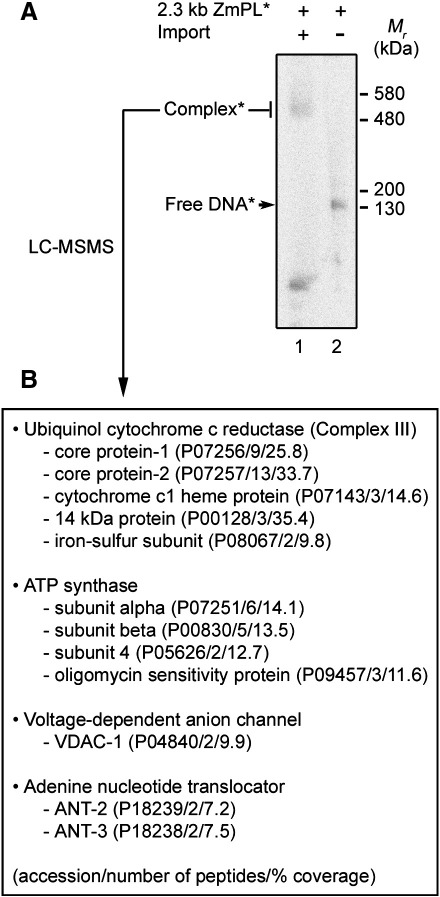
Upon native analysis of mitochondrial import samples the DNA is recovered in complexes likely to contain the VDAC. (A) BN-PAGE analysis. A standard DNA import assay was run with labeled maize 2.3 kb plasmid (2.3 kb ZmPL⁎) and mitochondria isolated from the parental yeast strain M3. Following DNase-I treatment, the mitochondrial sample was lysed and the membrane fraction was submitted to electrophoresis on a Blue Native polyacrylamide gel (see Materials and methods) so as to fractionate the protein complexes (lane 1); an aliquote of the labeled DNA probe was run in parallel on the same gel (lane 2). The radioactivity was revealed upon exposure of the gel to an imaging plate. Migration of the DNA probe alone is indicated (Free DNA⁎). (B) Mass spectrometry analysis. The region corresponding to the high molecular weight complex(es) associated with labeled DNA (Complex⁎) was excised from the Blue Native gel and its protein content was analysed by nano-LC-MSMS mass spectrometry. The proteins identified are listed, with the accession number, the number of matching peptides and the percent of coverage given in brackets in the mentioned order.

As a preliminary approach to identify candidate partners of the VDAC in the DNA uptake process, we reasoned that some radiolabeled DNA substrate would maintain an interaction with complexes involved in its recognition and mitochondrial translocation, allowing the detection and identification of these complexes by proteomic analysis. For that purpose, the mitochondrial membrane fraction was recovered from standard DNA import samples and solubilised with the soft detergent digitonin, so as to release native protein complexes. The latter were separated by electrophoresis on Blue Native polyacrylamide gels (BN-PAGE). Exposure of the Blue Native gel indeed revealed the incorporation of the radioactive DNA into a high molecular weight structure in the range of 500 kDa (Fig. 5A). A gel sample matching the putative complex was recovered and the retained DNA was phenol extracted and analysed on an agarose gel (not shown). It turned out that, likely due to remaining nucleases, the initial probe had been cleaved during the experimental steps run in native conditions, so that fragments in the range of 100 nucleotides were actually retained and protected in the high molecular weight complex. The influence of the DNA itself on the migration of the latter was thus limited.

Mass spectrometry analysis of the protein content in the area of the Blue Native gel matching the radioactive complex identified a number of subunits of complex III and of the ATP synthase, *i.e.* proteins from major and abundant high molecular weight mitochondrial components (Fig. 5B). However, in line with the above data, nano-LC-MSMS also detected the VDAC-1 (Fig. 5B). Migration of this polypeptide in the upper part of the gel was only understandable through its presence in a large structure and suggested that the VDAC may engage into high molecular weight complexes which interact with the DNA. Finally, the gel area matching the radioactive complex contained a further protein, the adenine nucleotide translocator (ANT) (Fig. 5B). It can be argued that the ANT and the phosphate carrier have been proposed to associate with the ATP synthase to form the ATP synthasome [30,31], which could explain the presence of the ANT in the high molecular weight fraction. Still, the identification of the ANT in the same fraction as the VDAC and the DNA is of interest, as a role of this carrier in DNA translocation was suggested by previous assays showing a strong inhibition of DNA uptake into plant mitochondria in the presence of the ANT ligand ADP or the inhibitor atractyloside [16]. These data altogether make the ANT a relevant candidate for a genetic analysis with *S. cerevisiae* mutants. However, carboxyatractyloside had no effect on DNA import into mammalian mitochondria [17], highlighting a potential limitation of a general approach, *i.e.* significant differences may exist between organisms in the mitochondrial DNA import mechanism.

### 3.5 Mutation of both VDAC genes reprogrammes mitochondrial membrane composition and maintains DNA import competence

**Fig. 6:**
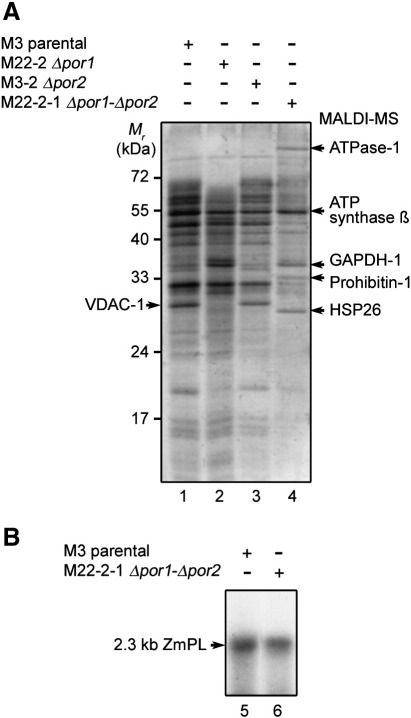
Mitochondria from the yeast double VDAC mutant retain competence for DNA uptake. (A) Mitochondrial membrane protein pattern analysis. Mitochondrial membrane proteins from the parental yeast strain M3, the M22-2 *Δpor1* strain, the M3-2 *Δpor2* strain and the M22-2-1 *Δpor1*–*Δpor2* strain were fractionated by SDS-PAGE and stained with Coomassie Blue. ATPase-1 (accession P05030/Mascot score 112), ATP synthase subunit β (accession P00830/Mascot score 179), glyceraldehyde-3′-phosphate dehydrogenase-1 (GAPDH-1; accession P00360/Mascot score 305), prohibitin-1 (accession P40961/Mascot score 76) and the 26 kDa heat shock protein (HSP26; accession P15992/Mascot score 214) indicated on the right side of the panel were identified by MALDI-TOF mass spectrometry analysis after excision of the corresponding polyacrylamide slices from lane 4 of the gel and in-gel trypsin digestion. VDAC-1 indicated on the left side of the panel was identified by western blot analysis as in Fig. 4B following fractionation of the same samples on an identical gel. (B) DNA import assays. Labeled maize 2.3 kb plasmid (2.3 kb ZmPL) was incubated for 40 min at 30 °C in standard DNA import conditions with mitochondria isolated from the parental yeast strain M3 or from the M22-2-1 *por1*–*Δpor2* strain. Following DNase-I digestion, uptake was analysed as in Fig. 1A. Migration of the incorporated substrate is indicated (2.3 kb ZmPL).

Assaying mitochondria from the *Δpor1*–*Δpor2* double mutant strain M22-2-1 showed the limits of the yeast genetic approach, as it seems that *S. cerevisiae* cells can in some cases develop substitution strategies for insufficient or deleted mitochondrial functions. Deletion of both VDAC genes at the same time led to substantial alteration of the mitochondrial membrane protein composition, as observed upon 1D SDS-PAGE (Fig. 6A). Whereas the membrane protein profile generated by mitochondria from the *Δpor1* or *Δpor2* mutant remained close to the control (Fig. 6A, lanes 1 to 3), the level of most of these polypeptides was very low in the profile corresponding to the organelles from the *Δpor1*–*Δpor2* double mutant (Fig. 6A, lane 4). Instead, three other proteins became predominant, namely HSP26, prohibitin-1 and ATPase-1, as identified by mass spectrometry. The level of ATP synthase subunit β remained unchanged. HSP26 is the principal small heat shock protein of *S. cerevisiae* [32] and forms shell-like particles [33,34]. Its activity is temperature dependent and controlled by the rearrangement of a thermosensor domain [35]. Prohibitins are believed to be chaperones but can form membrane-bound ring complexes associated with the mitochondrial inner membrane [36]. ATPase-1 (*e.g.* [37]) was previously found associated mainly with the plasma membrane but also with other subcellular compartments, including mitochondria [38]. It is a proton pump and the gradient it generates drives the transport of nutrients by H^+^-symport.

These observations established that the *por1*/*por2* double knockout caused substantial nuclear reprogramming destined to maintain sustainable mitochondrial transport capability. Such double mutants were indeed shown earlier to be viable and able to grow on glycerol at 30 °C [19], implying that, in the absence of VDAC expression, they had developed compensatory pathway(s) for the transport of solutes through the outer membrane. Strikingly, these alternative mechanisms were also able to ensure DNA translocation, as the DNA import capacity of the mitochondria from the *Δpor1*–*Δpor2* double mutant was almost equivalent to that of organelles from the parental strain (Fig. 6B). However, it was obvious from the above data that this DNA transport process was not representative of the regular DNA import pathway we wish to decipher. Notably, previous studies demonstrated that upon deletion or insufficient permeability of VDAC-1, the TOM complex (translocase of the outer membrane) partially substitutes for the VDAC channel in the transport of external solutes, such as the dinucleotide NADH, through the outer membrane [39]. The phenomenon includes upregulation of TOM proteins. Drawing a parallel with existing data on the mitochondrial import of cytosolic transfer RNAs (tRNAs), this could be an interesting idea to consider for an alternative pathway of DNA uptake into mitochondria from the *Δpor1*–*Δpor2* double mutant. Indeed, natural import of cytosolic tRNA^Lys^ into *S. cerevisiae* mitochondria requires a functional protein import pathway [40] and mitochondrial import of tRNAs in plants is likely to involve some of the TOM proteins, namely TOM20 and TOM40, besides the VDAC [28].

## 4 Conclusion

Why is it so important to understand natural competence, especially when all the experiments have to date been performed with isolated organelles? Transfection of plant and mammalian mitochondria is an extremely desirable goal for many reasons, both for pure and applied biology. The expression of imported foreign DNA from within the mitochondrion in a higher eukaryote cell has yet to be demonstrated. There are many difficulties that need to be addressed when considering effective mitochondrial transfection. One of these obstacles is considered to be the uptake of DNA into mitochondria. Therefore, even if one was able to design a mitochondrial mini-vector that should be retained and expressed in mitochondria, initial targeting and uptake of the DNA has always been seen to be a major difficulty in situ. The observation that mitochondria have retained an ability to import naked DNA makes this difficulty resolvable. Results now consistently establish that isolated mitochondria can import DNA fragments of over 10 kb in size. Imported DNA acts as a template for DNA and RNA synthesis in plant and mammalian mitochondria [16,17]. Further, in mammalian mitochondria the transcript generated from the exogenous template has been shown to be processed and matured. However, to delineate the process and to identify the important players are essential. It is especially striking that, although they may have an influence in some cases, neither membrane potential nor exogenous ATP are strictly required, begging the question of how the import of such a large polyanion can be driven.

Perhaps the greatest challenge is to understand how DNA can cross the impenetrable barrier of the inner membrane and to determine what the key factors in this process are. Classic mitochondrial solute transporters that are involved in nucleotide transport would seem to be too small to facilitate polynucleotide import. However, this is a very large family of proteins, many of which have yet to be characterised (for review see [41]). Impressive advances have been made in reconstituting several of these proteins [42]. Now that natural competence of yeast mitochondria has been shown, the large bank of *S. cerevisiae* strains carrying mutations in these family members makes it possible to screen for deficiencies in DNA import and hasten our identification of its key players, keeping in mind the limits highlighted in the present work.
